# Randomized double-blind clinical trial comparing safety and efficacy of the biosimilar BCD-021 with reference bevacizumab

**DOI:** 10.1186/s12885-022-09243-7

**Published:** 2022-02-01

**Authors:** Daniil L. Stroyakovskiy, Natalya V. Fadeeva, Marina P. Matrosova, Konstantin G. Shelepen, Grigoriy A. Adamchuk, Bodhisatta Roy, Rajnish Nagarkar, Mahesh Kalloli, Daria Zhuravleva, Georgiy D. Voevodin, Mariya S. Shustova, Fedor Kryukov

**Affiliations:** 1 Moscow City Oncology Hospital No. 62, Moscow, Russian Federation; 2 Chelyabinsk Regional Clinical Center for Oncology and Nuclear Medicine, Chelyabinsk, Russian Federation; 3 Nizhny Novgorod Regional Clinical Oncological Dispensary, Nizhny Novgorod, Russian Federation; 4 Brest Regional Clinical Cancer Dispensary, Brest, Republic of Belarus; 5 Kryviyi Rih Oncology Dispensary, Kryvyi Rih, Ukraine; 6 Netaji Subhas Chandra Bose Cancer Research Institute, Kolkata, India; 7 HCG Manavata Cancer Centre, Nashik, India; 8 KLE Hospital & Research Institute, Belgaum, India; 9 JSC BIOCAD, Saint Petersburg, Russian Federation

## Abstract

**Background:**

BCD-021 is a bevacizumab biosimilar which was shown to be equivalent to reference bevacizumab in a wide panel of physicochemical studies as well as preclinical studies *in vitro* and *in vivo*. International multicenter phase III clinical trial was conducted to compare efficacy and safety of BCD-021 and reference bevacizumab in combination with paclitaxel and carboplatin in a first-line treatment of inoperable or advanced non-squamous non-small-cell lung cancer (NSCLC).

**Methods:**

Patients with no previous treatment for advanced non-squamous NSCLC were randomly assigned 3:2 to BCD-021 or reference bevacizumab and were treated with bevacizumab + paclitaxel + carboplatin. Therapy continued for 6 cycles (every 3 weeks), until progression of the disease or unbearable toxicity. The primary study endpoint was the overall response rate. The study goal was to prove the equivalent efficacy of BCD-021 and reference bevacizumab. Equivalence margins for 95% CI for the difference in the overall response rates were set at [-18%; 18%], for 90% CI for the ratio of overall response rate were set at [67%; 150%].

**Results:**

In total 357 patients were enrolled in the study, 212 in the BCD-021 group and 145 in the reference bevacizumab group. The ORR was 34.63% in the BCD-022 group and 33.82% in the reference bevacizumab group. Limits of 95% CI for the difference in overall response rates between the groups were [-9.47%; 11.09%]. Limits of 90% CI for the ratio of overall response rate between the groups were [79.6%; 131.73%]. For both approaches CI lied within predetermined equivalence margins. Profile of adverse events (AEs) was similar between the groups (any AEs were reported in 86.89% of patients in BCD-021 group and 89.05% of patients in reference group). No unexpected adverse reactions were reported throughout the study. No statistically significant differences regarding anti-drug antibody occurrence rate was found between BCD-022 (n=4; 1.96%) and comparator (n=5; 3.65%). Both drug products showed low occurrence rate and short life of anti-bevacizumab antibodies. Pharmacokinetics assessment after 1^st^ and 6^th^ study drug injection also demonstrated equivalent PK parameters by all outcome measures.

**Conclusions:**

Thus, the results of this study demonstrated therapeutic equivalence of bevacizumab biosimilar BCD-021 and referent bevacizumab drug.

**Trial registration:**

The trial was registered with ClinicalTrials.gov (Study Number NCT01763645, date of registration 09/01/2013).

**Supplementary Information:**

The online version contains supplementary material available at 10.1186/s12885-022-09243-7.

## Introduction

Bevacizumab is a humanized monoclonal antibody that selectively binds to the vascular endothelial growth factor (VEGF). Bevacizumab blocks interaction of VEGF with its receptors on the cell surface resulting in a suppression of tumor blood vessels growth and inhibition of tumor growth [1].

It is shown that the use of bevacizumab at a dose range of 7.5-15 mg/kg in first-line treatment of unresectable, recurrent, or advanced non-squamous non-small cell lung cancer (NSCLC) with combination chemotherapy including platinum agents leads to a significant increase in overall survival (OS), time to progression (TTP), and overall response rate (ORR) [2].

BCD-021 is a bevacizumab biosimilar which is developed and manufactured by JSC BIOCAD (Russian Federation). The complex of *in vitro* and *in vivo* preclinical studies including studies in primates showed that physicochemical, toxic, pharmacokinetic and pharmacodynamic properties of BCD-021 are equivalent to those of referent bevacizumab [3, 4].

### Objectives

The BCD-021-02 study tested the hypothesis of the equivalence of BCD-021 (bevacizumab by JSC BIOCAD, Russia) and Avastin (reference bevacizumab by F. Hoffmann-La Roche Ltd., Switzerland), both in combination with paclitaxel and carboplatin, in a first-line treatment of unresectable or advanced non-squamous NSCLC. The objectives of the study were to evaluate efficacy, safety and pharmacokinetics of BCD-021 compared with reference bevacizumab by 1. overall response rate and other efficacy parameters; 2. incidence and severity of adverse events; 3. serum concentration after the first and multiple bevacizumab administration; 4. incidence and concentration of anti-bevacizumab antibodies.

### Trial Design

This Phase III study was approved by the independent ethics committees including local independent committees at all participated study sites and performed in accordance with the ethical principles set forth in the World Medical Association Declaration of Helsinki “Ethical Principles for Medical Research Involving Human Subjects” or comparable national ethical standards, and International Conference on Harmonization Good Clinical Practice guidelines. All subjects provided written informed consent before starting screening procedures. The study was international, multicenter, double-blind, randomized, two-arm, parallel-group trial comparing BCD-021 with the reference bevacizumab.

The first subject was enrolled in the study on 27 October 2012. A total of 357 were randomized in the study including 219 subjects in Indian study sites. In total 56 study sites enrolled subjects. Study sites were in four countries: 20 in Russia, 6 in Ukraine, 1 in Belarus, 29 in India. The trial was registered with ClinicalTrials.gov (Study Number NCT01763645, date of registration 09/01/2013). Clinical study report date is 22 June 2020.

### Participants

The trial included males and females 18-75 years of age with advanced non-squamous NSCLC. To be enrolled subject must have had at least one measurable lesion according to RECIST 1.1 on CT scan; ECOG score 0-2; life expectancy of at least 12 weeks. Exclusion criteria encompassed a number of medical conditions, including proven coagulopathy and clinically significant hemorrhage in the past; a history or presence of hypersensitivity; cardiovascular system pathology (CHF stage III-IV according to NYHA classification); uncontrolled hypertension; acute or active chronic infections; unstable central nervous (CNS) metastases or other malignancies, with the exclusion of radically treated basal cell carcinoma of skin or cervical cancer *in situ*. Previous major surgery must have been completed at least 28 days prior randomization. Any previous anticancer therapy for metastatic NSCLC was recognized as exclusion criteria.

### Randomization

After completion of 28-days screening period eligible subjects were centrally randomized into 2 treatment groups to receive either BCD-021 or reference bevacizumab (ratio 3:2, resp.). Randomized assignment was stratified according to performance status (ECOG 0-1 or 2), CNS metastases (present/not present), and NSCLC stage (IIIb/IV).

Since the study had a double-blind design, BCD-021 and reference bevacizumab had equivalent labeling and secondary package. Neither drug name nor manufacturer were indicated on primary or secondary package. Packages of investigational drug and comparator could be identified only by the batch number.

### Interventions

**Fig. 1:**
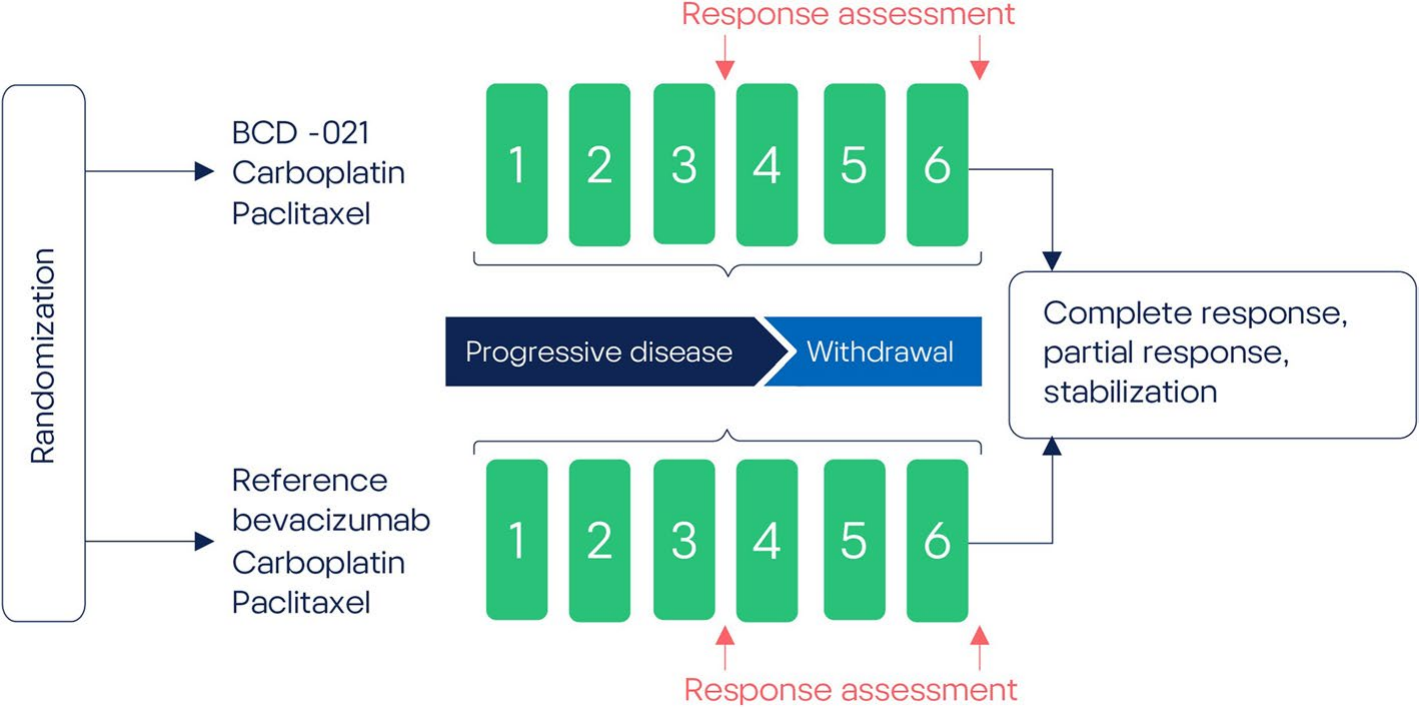
BCD-021-2 study design

Patients were treated with BCD-021 or reference bevacizumab at a dose of 15 mg/kg every 3 weeks, + paclitaxel 175 mg/m^2^ every 3 weeks, + carboplatin AUC 6 mg/ml×min every 3 weeks. Therapy continued for 6 cycles (every 3 weeks) until progression of the disease or unbearable toxicity whichever occurred first (Fig. 1). Therapy was administered as an intravenous infusion; infusion speed was corrected according to the scheme provided in the reference drug label. Premedication was mandatory before investigational treatment including glucocorticoid (dexamethasone), diphenhydramine (or its equivalent) and cimetidine (or ranitidine). Bevacizumab dose correction was not permitted. Paclitaxel and carboplatin dose adjustment was allowed according to the scheme provided in drug label. After the planned 6 cycles of therapy, subjects with complete or partial response or stable disease by the decision of Investigator were proceeded to the maintenance therapy period to receive unblinded maintenance therapy with BCD-021 (until disease progression or unbearable adverse events). No other therapies (e.g. surgery or radiation therapy) are used in the population with advanced NSCLC, except for those used per BCD-021-2 Protocol.

### Study procedures

Contrast-enhanced computed tomography (CT) for the efficacy assessment was performed within 28 days before random assignment (baseline), then after 3 therapy cycles and after 6 therapy cycles. The tumor response was assessed by central review based on the results of CT scan with contrast and using RECIST 1.1 criteria [5]. In case of primary registration of either complete or partial response, a confirmatory CT scan was made 4 weeks later.

To assess treatment safety on each visit data on adverse events were collected and vital signs were measured (body temperature, heart rate, respiration rate, blood pressure); also, throughout the study complete blood count, blood chemistry, urinalysis, ECG and Echo were controlled.

Blood samples for immunogenicity assessment were collected prior to the first bevacizumab administration (baseline), then 14±1, 64±2, 127±2 and 154±2 days after the last bevacizumab administration. As per protocol, the screening test was performed to detect the presence of binding antibodies (BAb) in subject’s blood, followed by the confirmatory analysis. If binding antibodies were found, the test for neutralizing antibodies (NAb) was performed. Presence and concentrations of anti-bevacizumab antibodies were determined centrally using ELISA. Detection of neutralizing anti-bevacizumab antibodies (neutralizing potency of binding antibodies) was performed by a validated antiproliferative test in BT-474 cell culture.

Blood samples for pharmacokinetics analysis were obtained on day 1 immediately before the start of the 1st bevacizumab infusion, then 1.5, 3 (±15 min), 4.5 (±15 min), 6 (±15 min), 24±1, 96±8, 168±8, 336±8 and 504±8 hours after the first administration immediately before subsequent infusions. Blood samples were collected immediately prior to each bevacizumab administration and in 504±8 h after the 6^th^ drug administration. Additionally, to study pharmacokinetics at steady state blood samples were collected immediately before start of the 6^th^ study or reference drug administration and 1.5, 3 (±15 min), 4.5 (±15 min), 6 (±15 min), 24±1, 96±8, 168±8, 336±8, 504±8 h (21 days) after the 6^th^ bevacizumab infusion.

### Study endpoints

The primary study endpoint was overall response rate (cumulative rate of complete and partial responses) in advanced non-squamous NSCLC subjects after receiving up to 6 cycles (18 weeks) of bevacizumab + paclitaxel + carboplatin therapy. The treatment response was assessed by CT scans according to RECIST 1.1 criteria and was centrally evaluated by an independent specialist.

Secondary efficacy endpoints were partial response and complete response rates, rates of stable disease and progressive disease.

Safety endpoints included incidence and types of adverse events (AEs), study therapy-related adverse events, treatment withdrawal due to adverse events. All AEs were calculated using the maximum severity grade reported throughout the study. Serious adverse events (SAEs) were registered in the screening period as well. The severity of AEs was assessed according to the Common Terminology Criteria for Adverse Events (CTCAE v.4.03). and coded using MedDRA (version 21.1) preferred terms.

Immunogenicity endpoints included incidence of antidrug antibody formation and neutralizing activity of detected antibodies. Pharmacokinetics endpoints included area under the serum concentration-time curve (AUC), maximum serum concentration (C_max_), time to maximum serum concentration (T_max_) and trough concentration (C_trough_) of bevacizumab during 6 cycles.

### Statistical analysis

According to the ICH E10 Guideline the margin generally should not exceed difference between active control and placebo (or standard therapy) based on past experience in placebo-controlled trials (or active controlled studies) of adequate design under conditions similar to those planned for the new trial [6]. The sample size was calculated using the following variables: 2-sided α=0.05, study power of 80%. To define an equivalence margin historical data were reviewed:

- the difference between ORR of bevacizumab with chemotherapy (37.7%) and chemotherapy alone (19.3%) according to FDA’s meta-analysis results [7],
- the difference between maximum (56.2%) and minimum (32.4%) ORR of bevacizumab with chemotherapy for all studies from this meta-analysis [7].

Thus, in current study δ (an equivalence margin) should not be higher than 18.4%. It was hypothesized that 95% CI for the difference between ORR in the BCD-021 group and in the reference bevacizumab group will be within the limits of -18% to 18%, i.e. equivalence criterion δ = 0.18. Thus, it was necessary to enroll 104 subjects for control group and 156 subjects for the test group; therefore, with a sample size of 260 subjects, the study had 80% power to reject the null hypothesis at α = 0.05.

Alternatively, similar sample size can be calculated using the ratio of response rates. For these purposes we assumed that if the ratio of response rates (risk ratio, RR) for BCD-021 to reference bevacizumab is more than 0.67 and less than 1.5, the difference between these drugs can be considered clinically insignificant. As the equivalence margin we use upper boundary of 95% CI (CI: [0.44; 0.67]) for RR for the dose 15 mg/kg from random-effects model meta-analysis [2]. Thus, the lower equivalence margin was set as δ_1_ = 0.67. Upper margin was set as δ_2_ = 1/ δ_1_ ≈ 1.5. Thus, the necessary sample size is 112 subjects for control group and 168 subjects for test group; therefore, total sample size in trial is 280 subjects.

Summarizing all said above, sample size of at least 280 subjects is sufficient to prove that the efficacy of bevacizumab biosimilar (JSC BIOCAD) is equivalent to the efficacy of the referent bevacizumab with 80% power by using ORR difference with margin [-0.18, 0.18] as well as by using ORR ratio with margin [0.67; 1.5].

*The efficacy analysis* was performed in “modified intention-to-treat” population (mITT, subjects who received at least 1 infusion) if the assessment of response was possible (n = 341).

*The safety analysis* included all data from all randomized subjects who received at least one dose of study therapy (n= 343).

Analysis of main *pharmacokinetic parameters* of bevacizumab at the first cycle of therapy was performed in subjects who received at least one bevacizumab infusion and had no more than one missed PK serum sample (n=300). Analysis of main pharmacokinetics parameters of bevacizumab at the 6^th^ cycle of therapy was performed in subjects who received the 6th bevacizumab infusion and had not more than one missed sample during the 6th therapy cycle (n=97). Analysis of trough concentrations of bevacizumab was performed in subjects who received all 6 cycles of therapy (6 bevacizumab infusions) and missed not more than 1 PK sampling before every bevacizumab infusion (n=161).

*Immunogenicity analysis* was performed in subjects who received at least one infusion of study therapy with at least one post-baseline blood sample for immunogenicity assessment available (n=343).

For the normally distributed data two-sample t-test and analysis of variances were used.

For the non-normally distributed data Mann-Whitney test, Wilcoxon test and Kruskal-Wallis test were implemented.

Two or more independent groups were compared for the quantitative parameters using ANOVA (one-way analysis of variance), Kruskal-Wallis test and median test.

Processing of categorical data was performed using frequency (one-way) tables, cross (multi-way) tables, exact Fisher test, equality of frequency test, and Pearson’s chi-square test (Yates-corrected test was used for cross tables 2×2). Percentages or proportions were used to describe categorical data.

Feasibility of using different statistical methods was evaluated after completion of the data collection, as the distribution pattern and sample homogeneity were not known in advance.

Statistical analysis was performed using SAS 9.4 software and R 4.1.1.

## Results

**Fig. 2:**
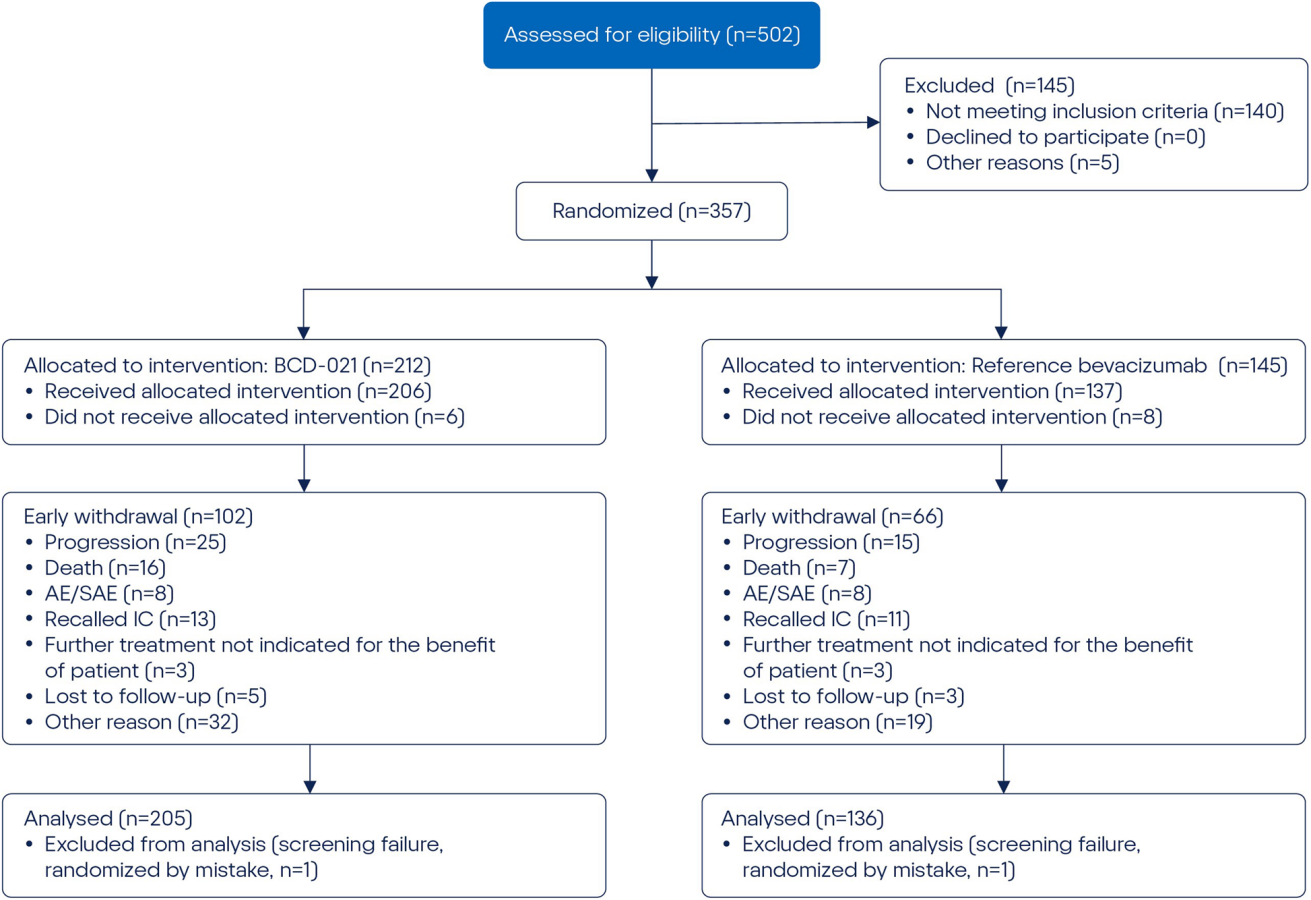
Disposition of subjects by study groups and reasons for withdrawal

For subjects in all sites early withdrawal was reported in 48.11% (102/212) of subjects in BCD-021 group and 45.52% (66/145) in reference bevacizumab group. The most frequent reason for early withdrawal was progression of disease during the study: 11.79% (25/212) and 10.34% (15/145) in BCD-021 and reference bevacizumab groups, respectively (Fig. 2).

For pooled ITT population most subjects had Asian race (67.32% and 52.21% for groups that received BCD-021 and reference bevacizumab respectively) and most subjects were male (69.27% and 64.71% for groups that received BCD-021 and reference bevacizumab respectively). Most subjects had ECOG = 1 (75.12% and 74.26% for groups that received BCD-021 and reference bevacizumab respectively). Most subjects had Stage IV disease (86.34% and 82.35%) and most subjects had adenocarcinoma as a tumor histology (98.54% and 98.53% for groups that received BCD-021 and reference bevacizumab respectively) (Table 1).

**Table 1:** Demographic characteristics and baseline characteristics of study disease (Pooled ITT Population)

Parameter	BCD-021 (N = 205) n (%)	Reference bevacizumab (N = 136) n (%)
Race Asian	138 (67.32)	71 (52.21)
Black	0	1 (0.74)
White	67 (32.68)	64 (47.06)
Sex		
Male	142 (69.27)	88 (64.71)
Female	63 (30.73)	48 (35.29)
Childbearing potential (Female)		
Yes	12 (5.85)	8 (5.88)
ECOG score		
0	41 (20.00)	25 (18.38)
1	154 (75.12)	101 (74.26)
2	10 (4.88)	10 (7.35)
Allergy		
Yes	2 (0.98)	5 (3.68)
Stage		
III	5 (2.44)	8 (5.88)
IIIB	23 (11.22)	16 (11.76)
IV	177 (86.34)	112 (82.35)
Histological type		
Adenocarcinoma	202 (98.54)	134 (98.53)
Bronchoalveolar	3 (1.46)	1 (0.74)
Large cell	0	1 (0.74)

N: number of subjects in population; n: number of observations; Percents is calculated: [100 x (n / N)]

The groups were similar in all demographic and other baseline characteristics except the race in pooled ITT population: there were more subjects with reference bevacizumab race in BCD-021 group compared to reference bevacizumab group (p=0.005, Pearson’s chi-squared test). Also, there were more white subjects in reference bevacizumab group compared to BCD-021 group (p=0.0075, Pearson’s chi-squared test). Differences in mean height and weight in ethnic groups are presented: mean weight was 56.6 (±11.4) and 74.5 (±13.5) kg, respectively, for Indian and non-Indian population. Mean height was 161 (±9) and 169 (±8) cm, respectively, for Indian and non-Indian population.

### Efficacy

According to CT-scan results, the overall response rate was 34.63% (71/205) and 33.82% (46/136) in BCD-021 and reference bevacizumab groups, respectively (Table 2).

**Table 2:** Efficacy endpoints assessment results (pooled mITT population)

Parameter	BCD-021 (N=205) N% (95% CI)	Reference bevacizumab (N=136) N% (95% CI)	p-value
**Primary outcome measure**			
ORR (confirmed)	7134.63 (28.46 - 41.38)	4633.82 (26.41 - 42.12)	0.8773^2^
**Secondary outcome measure**			
CR rate (confirmed)	31.46 (0.5 - 4.21)	10.74 (0.13 - 4.05)	1.0000^1^
PR rate (confirmed)	6833.17 (27.09 - 39.87)	4533.09 (25.74 - 41.37)	0.9874^2^
PR rate (unconfirmed)	52.44 (1.05 - 5.58)	42.94 (1.15 - 7.32)	0.7458^1^
Stable disease	6531.71 (25.72 - 38.36)	4633.82 (26.41 - 42.12)	0.6830^2^
Progressive disease	2713.17 (9.21 - 18.48)	1511.03 (6.8 - 17.4)	0.5557^2^

Note: ORR – overall response rate; CR – complete response; PR – partial response; N = number of subjects in Analysis Set; n = number of subjects with responses. ^1^ Fisher's exact test, ^2^ Pearson's chi-squared test

The difference in ORR between BCD-021 and reference bevacizumab groups in pooled mITT population was 0.81%, with 95% CI for the difference: [-9.47%; 11.09%], i.e. within pre-determined equivalence margin [-18%; 18%].

The result of 90% CI calculation for the ratio of overall response rate (risk ratio, RR) between BCD-021 and reference bevacizumab groups in pooled mITT population was [79.6%; 131.73%]; i.e. lies within the predefined range of equivalence margin [67%; 150%].

For both approaches CI was completely within the predefined range of clinically insignificant difference, thus equivalent efficacy of BCD-021 and reference bevacizumab was established.

**Fig. 3:**
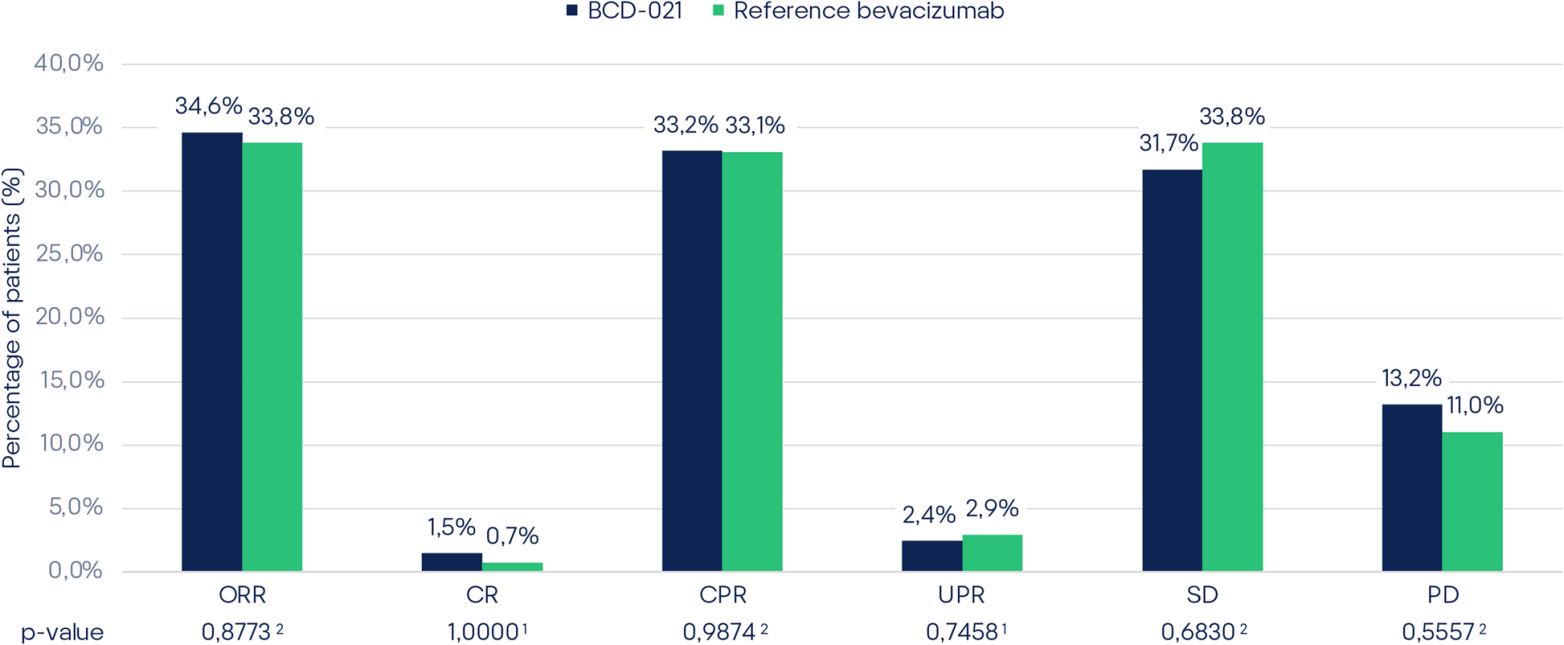
Efficacy endpoint assessment results (pooled mITT population). Note: ORR — overall response rate; CR — complete response; CPR — confirmed complete response; UPR — unconfirmed partial response; SD — stable disease; PR — progressive disease. Note: 1 — Fisher’s exact test; 2 — Pearson chi-square test

Comparison of other efficacy assessment parameters (secondary outcome measures) did not show any statistically significant differences between the study groups in pooled mITT population (Fig. 3).

**Sensitivity analysis** was done by the same 90% CI calculation for relative risk between BCD-021 and reference bevacizumab in three analysis sets (randomized subjects, intent-to-treat, per protocol population) to compare CI boundaries with pre-defined equivalence margins (Table 3). Given that all sensitivity analyses confirmed the primary analyses outcomes in mITT population, the robustness in veracity of the latter is confirmed.

**Table 3:** Sensitivity analysis for overall response rate

Analysis Set	Treatment	n/N	(%)	Relative Risk	90% CI
Randomized	BCD-021 (N=212)	71/212	33.49	1.0557	[81.79%; 136.26%]
	reference bevacizumab (N=145)	46/145	31.72		
ITT	BCD-021 (N=205)	71/205	34.63	1.024	[79.6%; 131.73%]
	reference bevacizumab (N=136)	46/136	33.82		
PP	BCD-021 (N=163)	71/163	43.56	1.0133	[80.2%; 128.04%]
	reference bevacizumab (N=107)	46/107	42.99		

Note: ITT- intent to treat, PP- per protocol, N = number of subjects in reference bevacizumab Set, n = number of subjects with responses.

**Post-hoc stratified analysis** was done for ethnic group as the stratification factor with levels “Indian” and “Not-Indian” in mITT and PP populations. The non-significant p-values (>0.6) of the Breslow-Day test for both mITT and PP correspondingly indicate no significant difference between ethnic groups in the odds ratios.

### Safety

In the pooled safety population treatment discontinuation due to AEs/SAEs was reported in 1.75% (6/343) of the subjects: 1.94% (4/206) subjects from BCD-021 group and 1.46% (2/137) subjects in the comparator group (p = 1.0000).

Study groups had no significant difference regarding the frequency of any SAE as well as no differences in frequency of SAEs related to the study therapy (р > 0.05).

In the pooled population SAE were registered in 12.54% (43/343) subjects: 13.59% (28/206) subjects from BCD-021 group and 10.95% (15/137) subjects from the comparator group (p = 0.4690). According to investigators, there were 2.92% (10/343) SAEs related to the study therapy: 3.40% (7/206) SAEs in BCD-021 and 2.19% (3/137) SAEs in comparator group (p = 0.7454) (Table 4).

**Table 4:** Safety endpoints (pooled safety population)

Deviation	BCD-021 (N = 206) n (%)	Reference bevacizumab (N = 137) n (%)	Total (N=343) n (%)	p-value
Any AE (including SAE)	188 (91.26)	128 (93.43)	316 (92.13)	0.4651^1^
AE Grade 3-5 (including SAE)	74 (35.92)	51 (37.23)	125 (36.44)	0.8059^1^
SAEs	28 (13.59)	15 (10.95)	43 (12.54)	0.4690^1^
Therapy-related SAE	7 (3.40)	3 (2.19)	10 (2.92)	0.7454^2^
Treatments discontinued due to AE/SAE	4 (1.94)	2 (1.46)	6 (1.75)	1.0000^2^
Deaths	14 (6.80)	8 (5.84)	22 (6.41)	0.7232^1^

Note: ^1^ - Pearson’s chi-suared test, ^2^ - Fisher’s exact test; Comparisons and calculation of the statistical significance of the differences between the groups BCD-021 and reference bevacizumab;

N: number of subjects; n: number of observations; Percent is calculated: [100 x (n / N)]

Generally, SAEs were associated with the underlying pathology, chemotherapy agents used in combination therapy or with other factors unrelated to the study therapy.

In total, during the study, 22 lethal outcomes were reported in pooled population: 6.80% (14/206) subjects from BCD-021 group and 5.84% (8/137) subjects from the comparator group, with no significant difference showed (р = 0.7232).

Thus, both the study drug BCD-021 and the reference bevacizumab were adequately tolerated by subjects throughout the entire study. No differences in safety profile with respect to the pre-determined safety endpoints were observed.

### Immunogenicity

Throughout the study neutralizing antibodies were detected in 9 subjects: 1.94% (4/206) from BCD-021 group and 3.65% (5/174) from comparator group (p = 0.4924).

Thus, no statistically significant differences regarding anti-drug antibody occurrence rate were found. The immunogenicity of BCD-021 is similar to that of reference bevacizumab; both drug products are characterized with low occurrence rate and short life of anti-bevacizumab antibodies.

### Pharmacokinetics

In *Indian population* the pharmacokinetic analysis at the first cycle of therapy, the mean C_max_ was 185.25 (±106.45) and 182.39 (±118.54) μg/mL, and AUC was 27786.61 (±13180.14) and 29271.17 (±15474.17) μg·h/mL, respectively, for study and reference bevacizumab. Mean T_max_ was achieved at 13.8 h (median: 4.5 h) and 13.6 h (median: 4.5 h), respectively, for study and reference bevacizumab. For the comparisons of study to reference bevacizumab, the 90 % CIs for the test-to-reference ratios of C_max_ and AUC were all within the bioequivalence window of 80.00–125.00 % (Table 5).

**Table 5:** Statistical comparison of pharmacokinetic parameters at the first cycle (*Indian population*)

Parameter	Geometric mean		90% CI
	BCD-021	Reference bevacizumab	
C_max_ (μg/ml)	157.34	153.48	87.58% - 120.01%
AUC (μg·h/ml)	24519.49	25849.40	82.72% - 108.78%

In the pharmacokinetic analysis at the sixth cycle of therapy, the mean C_max_ was 351.46 (±205.33) and 345.27 (±254.91) μg/mL, and AUC was 58949.16 (±32489.24) and 60137.29 (±42136.03) μg·h/mL, respectively, for study and reference bevacizumab. Mean T_max_ was achieved at 10.9 h (median: 6.0 h) and 17.5 h (median: 4.5 h), respectively, for study and reference bevacizumab. Statistically, mean C_max_, AUC, and T_max_ were comparable in both groups.

In the pharmacokinetic analysis of trough concentrations statistical comparison of concentrations of study and reference bevacizumab prior to each bevacizumab administration and at 504 h after the 6th drug administration demonstrated the absence of any significant differences between the study groups.

In *Non-Indian population* the pharmacokinetic analysis at the first cycle of therapy, the mean C_max_ was 459.25 (±216.51) and 452.64 (±183.03) μg/mL, and AUC was 62237.55 (±29468.90) and 65381.93 (±32161.78) μg·h/mL, respectively, for study and reference bevacizumab. Mean T_max_ was achieved at 6.1 h (median: 3.0 h) and 5.6 h (median: 3.0 h), respectively, for study and reference bevacizumab. For the comparisons of study and reference bevacizumab, the 90 % CIs for the test-to-reference ratios of C_max_ and AUC were within the bioequivalence window of 80.00–125.00 % (Table 6).

**Table 6:** Statistical comparison of pharmacokinetic parameters at the first cycle (*Non-Indian population)*

Parameter	Geometric mean		90% CI
	BCD-021	Reference bevacizumab	
C_max_ (μg/ml)	420.93	422.53	89.12% - 111.35%
AUC (μg·h/ml)	54556.29	57999.13	80.67% - 109.69%

In the pharmacokinetic analysis at the sixth cycle of therapy, the mean C_max_ was 418.45 (±106.98) and 453.22 (±157.48) μg/mL, and AUC was 107942.90 (±39924.57) and 118810.50 (±45122.83) μg·h/mL, respectively, for study and reference bevacizumab. Mean T_max_ was achieved at 7.0 h (median: 3.0 h) and 8.0 h (median: 4.5 h), respectively, for study and reference bevacizumab. Statistically, mean C_max_, AUC, and T_max_ were comparable in both groups.

In the pharmacokinetic analysis of trough concentrations statistical comparison of concentrations of study and reference bevacizumab prior to each bevacizumab administration and in 504 h after the 6th drug administration demonstrated the absence of any significant differences between the study groups.

## Overall Conclusion

The efficacy, safety and pharmacokinetics analysis has found no significant differences between BCD-021 and reference bevacizumab groups. The efficacy analysis showed the similar number of complete and partial responses in subjects who received BCD-021 (JSC BIOCAD, Russia) and Avastin (F. Hoffmann-La Roche Ltd., Switzerland). All sensitivity analyses confirmed the primary analyses outcomes in mITT population, the robustness in veracity of the latter is confirmed.

According to EMA «Guideline on the investigation of bioequivalence»: «*In parallel design studies, the treatment groups should be comparable in all known variables that may affect the pharmacokinetics of the active substance (e.g. age, body weight, sex, ethnic origin, smoking status, extensive/poor metabolic status). This is an essential pre-requisite to give validity to the results from such studies»* *[*8*].* Indian and non-Indian populations of subjects included in the analysis of pharmacokinetics were statistically different by weight and height. As such differences could affect the pharmacokinetics, analysis of pooled population data was not considered appropriate to give valid results.

## Discussion

The findings obtained from the present study are comparable to the literature data. The table below demonstrates ORR fluctuation in different studies of chemotherapy + bevacizumab: these four studies were Study E4599 [9], Study JO19907 [10], Study AVF0757 [11], and the AVAiL study . The control arm in three of the studies was paclitaxel plus carboplatin. Cisplatin plus gemcitabine was used in the AVAiL study. As shown below, despite the use of a different backbone chemotherapy regimen in AVAiL, the magnitude of the bevacizumab treatment effect was comparable to that observed in the other three studies (Table 7).

**Table 7:** Overall response rate fluctuation in different studies of chemotherapy + bevacizumab

Study	N (CT/BCT)*	CT ORR	BCT ORR	Risk Ratio
AVF0757 [11]	32/34	18.8%	32.4%	0.58
JO19907 [10]	59/121	33.9%	56.2%	0.60
AVAiL [12]	327/329	21.7%	34.7%	0.63
E4599 [9]	392/381	15.1%	34.9%	0.43
FDA’s meta-analysis [7]	810/865	19.3%	37.7%	0.53
BCD-021-2	NA/206	NA	34.6%	NA

*N: ITT population; CT: chemotherapy; BCT: bevacizumab with chemotherapy; NA: not applicable

As it clearly seen the inter-study treatment effect on ORR ranging from 32.4% to 56.2% in patients with NSCLC who received combination of bevacizumab with chemotherapy and does not exceed ORR difference between CT and BCT (18.4%) obtained in FDA’s meta-analysis [7]. It indicates that fluctuations of ORR in different trials in the same patient population exceed the chosen margin, therefore it can be considered clinically insignificant. Comparison with the literature data allows us to make an additional assertion of the validity of the results obtained

Biosimilar development is a complex stepwise process, implying comparative study of wide panel of parameters, including physicochemical properties, functional characteristics, efficacy, and safety [4]. Regulatory guidelines for biosimilar agents have been established, but they do not specify the best ways to adopt them into real-world practice. Integrating of biosimilars into clinical practice is a complex process with several stakeholders, which, however, could potentially help establish a better control over cancer therapy costs.

Extrapolation of a biosimilar to indications for which it was not tested during the clinical trial program is common practice in Europe. According to ЕМА «Guideline on similar biological medicinal products containing monoclonal antibodies – non-clinical and clinical issues»: *«Extrapolation of clinical efficacy and safety data to other indications of the reference mAb, not specifically studied during the clinical development of the biosimilar mAb, is possible based on the overall evidence of comparability provided from the comparability exercise and with adequate justification ”[*13*].* In the case of bevacizumab biosimilars, the active substance blocks interaction of VEGF with its receptors on the cell surface and has no different impact on the subjects in the tested and non-tested therapeutic indications. The therapeutic indication studied in BCD-021-02 trial is relevant to other indications in terms of efficacy, and the homogeneous representative population with advanced NSCLC is sensitive for differences in all relevant aspects of efficacy. As it was stated above, BCD-021 (Avegra, JSC BIOCAD, Russia) and reference bevacizumab (Avastin, F.Hoffmann-La Roche, Switzerland) are comparable in the efficacy when used in combination with paclitaxel and carboplatin in inoperable or advanced non-squamous non-small-cell lung cancer subjects. Therefore, no additional data is required for efficacy extrapolation of BCD-021 (JSC BIOCAD, Russia) to all the indications of bevacizumab.

The number of biosimilars has been increasing, with more marketed medicines expected over the next few years, providing cost-effective treatments to more subjects. Among the different clinical applications of biosimilar medicines, cancer treatment remains the main target area. The experience of application of bevacizumab biosimilar (JSC BIOCAD, Russia) in routine clinical practice for subjects with advanced NSCLC is gradually accumulated. Thus, a comprehensive pharmacovigilance study is going on, monitoring the marketed biosimilar, and providing more useful information to clinicians regarding the safety and efficacy of this medicine.

## Supplementary Information

**Additional file 1.**

## Data Availability

The datasets generated and/or analyzed during the current study are not publicly available due to containing information that could compromise research participants privacy and consent under Russian Federal Law No. 323-FZ and Russian Federal Law No. 61-FZ but are available from the corresponding author on reasonable request with the consent of the participants.

## Abbreviations

*AE*: Adverse Event
*ANOVA*: One-way analysis of variance
*AUC*: Area Under Curve
*AUMC*: Area Under the First Moment Curve
*BAb*: Binding Antibodies
*CHF*: Congestive Heart Failure
*CI*: Confidence Interval
*Cl*: Clearance
*C*_*max*_: Peak Serum Concentration
*CNS*: Central Nervous System
*CR*: Complete Response
*CT*: Computer Tomography
*CTCAE*: Common Terminology Criteria for Adverse Events
*ECG*: Electrocardiography
*ECOG*: Eastern Cooperative Oncology Group
*ELISA*: Enzyme-linked Immunosorbent Assay
*EMA*: European Medicines Agency
*ITT*: Intention-To-Treat
*JSC*: Joint Stock Company
*К*_*el*_: Elimination Rate Constant
*mITT*: Modified Intention-To-Treat
*NAb*: Neutralizing Antibodies
*NSCLC*: *Non-Small Cell Lung Cancer*
*NYHA*: New York Heart Association
*ORR*: Overall Response Rate
*PD*: Progressive Disease
*PK*: Pharmacokinetics
*PP*: Per Protocol
*PR*: Partial Response
*RECIST*: Response Evaluation Criteria in Solid Tumors
*SAE*: Serious Adverse Event
*SD*: Standard Deviation
*ST*: Stabilization
*T*_*1/2*_: Half-life Period
*T*_*max*_: Time to Reach Peak Serum Concentration
*V*_*d*_: Volume of Distribution
